# "The Hospital" Nursing Mirror

**Published:** 1901-06-15

**Authors:** 


"The Hospital" Nursing Mirror.

Being the Nursing Section of "The Hospital."

[Contributions for this Section of "The Hospital" should be addressed to the Editor, "The Hospital" Nursing Mirror, 28 & 29 Southampton Street Strand, London, W.C.]

### Notes on News from the Nursing World.

### HONOURS FOR ARMY SISTERS.

Up to Tuesday evening it was generally supposed that the nursing sisters who had been recommended for war medals would receive them from the hands of the King on Wednesday morning. The fact is, as our representative was informed at the War Office, that their names were omitted on account of the length of the ceremony. The following are the ladies on whom the honour is to be conferred:—Lady Sarah Wilson, Sisters A. Nixon, D. D. Tripp, C. K. E. Steet, L. E. V. Asman, K. L. T. Babb, A. A. Bowles, M. A. C. Grant, B. M. Hoare, I. S. Lambe, C. M. Theophilus, K. Ward, J. E. Skillman, A. D. Redstone (New Zealand), B. Huston (Queensland), and A. B. Stephenson (Australian Nursing Service). On Monday afternoon Matron A. Ward and Nursing Sister K. Neville, both of the West African Frontier Force, were introduced into the presence of the King and Queen, and were decorated by the King with the badge of the Royal Red Cross.

### AN ARMY SISTER ON MILITARY NURSING.

Concurring in the view that suggestions and criticism in regard to military nursing reform should come from within, and that sisters, military and civilian, who have worked under the Army system should have an opportunity to express their opinions and experiences, we print in another page the first of a couple of contributions by an Army sister of experience gained in military hospitals at home and abroad, in which the present position is diagnosed and various reforms are proposed. The articles, of course, only express the personal convictions of the writer, who this week puts the "Orderly" question forward as the crux of the whole matter. The excellent spirit in which she approaches the question may be gathered by her statement that the abolition of orderlies is impracticable so long as the possibility of war has to be reckoned with, while she insists that "some plan which will raise the standard of conduct, professional skill, and general intelligence among a body of men where conscientiousness and knowledge are essentials to the proper discharge of duty," is indispensable. The changes she suggests, and other points of great interest, will be dealt with next week.

### DEATH OF TWO WAR NURSES.

In addition to the death at Pretoria of Sister Gilbert, one of the nurses selected for duty by Queen Alexandra, that of Sister Webster, at Kroonstad, is also announced. Both nurses were victims of enteric fever. Miss Gilbert was at the London Hospital prior to her departure to the front, and is the second member of the staff who has lost her life in tending the sick and wounded soldiers.

### THE JUBILEE NURSES IN IRELAND.

It is satisfactory to find that persons of all creeds and parties at Mallow concur in the opinion that the work of the District Nursing Association has been most valuable. During April the superintendent of the Queen Victoria Jubilee Nurses for Ireland visited Mallow and expressed herself pleased with the inspection—a view in which Father Corbett and Dr. Sheehan entirely agree. This should not be without effect upon the movement which has been set on foot in the sister island to obtain support for the Women's Memorial to Queen Victoria. Although the services of the Jubilee Nurses, wherever they have been employed in Ireland, have been recognised, the fact that there are at present only 74 centres as compared with 280 in Scotland, indicates that there is a necessity to make more widely known the immense benefits derived by the poor from the presence among them of a trained district nurse.

### THE BRITISH NURSES' ASSOCIATION AND "PSEUDO-ANGELS."

A little difference of opinion as to the value of opposition to a cause was manifested at the annual meeting of the Royal British Nurses' Association. Mr. Fardon, alluding to the manner in which the Association had been attacked, observed that adverse criticism did not do the slightest harm; and Mrs. Dacre Craven, with less hesitancy, declared that a little of it was a very good thing. Sir James Crichton-Browne, on the other hand, said he did not admit that opposition was necessary. The vulgar abuse to which the Association has been subjected is certainly neither necessary nor justifiable, and the figures which Mr. Fardon was able to quote, showing that the nurses belonging to the Association earned £14,000 last year, indicate the growing success of the bureau. According to Sir James Crichton-Browne, the object of the Association should be to get rid of "pseudo-angels," whose more prosaic description is "inadequately trained nurses." In this direction there is plenty of scope for good work on the part of the organisation, and we hope that its efforts will be supported as strongly as they deserve.

### NURSES WANTED IN CAPE COLONY.

From Queenstown we learn that nurses are just now in great demand in some parts of Cape Colony. In many cases it is impossible to procure a trained nurse, and according to a well-known medical man in Queenstown, anyone who would settle in that town, or a town of similar size in the Colony, might easily earn from £200 to £250 per annum. The difficulty in the Colony is, it appears, to induce a nurse to stay in one place long enough to become thoroughly known. We hear of a nurse who has been sent for to four cases in one week lately. This certainly tends to prove the dearth of nurses—a dearth which will probably soon be remedied.

### HYSTERIA AND ENTERIC.

A Wolverhampton medical man who lately returned from South Africa tells a curious story of the person who was secretary of one of the hospitals at Bloemfontein. He states that after a number of Army nurses, of whom he was put in medical charge, had been at Bloemfontein three weeks, the secretary of the hospital said they were "hysterical." When this remark was reported to Dr. MacMunn by one of the sisters, he spoke to the wife of the Commander-in-Chief, and in an hour an explanation was demanded. The truth was that several of the nurses, instead of being merely hysterical, were in the first stage of enteric fever. Dr. MacMunn, speaking from his own personal knowledge, declared that the work of the nurses at the front had been magnificent.

### MR. BOND AND THE WESTMINSTER NURSES.

We learn that Mr. Thomas Bond, the well-known surgeon, whose death took place under very distressing circumstances last week, was attended during his illness by two trained nurses from the Private Nurses' Home connected with Westminster Hospital. His relations with the nursing staff of the hospital—with which he ceased to be connected about a year ago—were very happy; and our representative was informed by the matron that he was always very kind to them. The evidence at the inquest shows that no blame attaches to the nurse who was in attendance on Mr. Bond at the time of his suicide. Miss Truncheon gave her evidence with great clearness. From 11 P.M. until after 6 A.M. she did not leave her patient, and it was not until he seemed sound asleep, and she herself had been reading a book for ten minutes, that she ventured to quit the room to empty a basin. She stated that she had been absent "scarcely a minute," when she heard the rustling of a blind. At the funeral on Monday wreaths were sent from the matron and nursing staff of the Westminster Hospital, and from the private nurses.

### PORTSMOUTH WORKHOUSE INFIRMARY NURSES.

Last week the probationers who have either completed, or nearly completed, their training at Portsmouth Workhouse Infirmary were publicly congratulated by the Chairman of the Board of Guardians upon the manner in which they had acquitted themselves, and their success in passing an examination by Dr. Bryant, of Guy's Hospital. It will be recollected that the training school was only started three years ago, and the result of the experiment must be extremely gratifying to Dr. Knott, the medical superintendent, by whom they were trained. In fact, in his review of the work, Dr. Knott expresses the satisfaction he has derived from his labours. He truly says that "a workhouse infirmary affords abundant opportunities for the cultivation of high moral tone, tact, energy, sympathy, and intelligence." It is interesting to learn that the matron of Guy's Hospital, who has been over the infirmary, and signified the pleasure her visit afforded her, has intimated her readiness to take one of the certificated probationers from time to time, give her a year's free training in the surgical wards of Guy's, and then take her on the private nursing staff. There is no doubt that as the standard of training which obtains in the school at Portsmouth Infirmary becomes known it will attract some of the well-educated young women who desire to embark on a nursing career, and that the Board of Guardians will reap the reward of their wisdom by obtaining an ample supply of capable charge nurses.

### A LOSS TO THE PENSION FUND.

We regret to record the death of Mr. Gerrard Norman, who, from the establishment of the Royal National Pension Fund until his death, had been a most useful and active member of the Council, at the meetings of which he was a most regular attendant. Mr. Norman will be missed in other circles of useful work also. He was on the Committee of Westminster Hospital, where his deep interest and useful assistance was much appreciated. There are few men who have given their personal service to philanthropy more generously and in a quiet unostentatious manner than Mr. Norman.

### AN EXAMPLE TO IMITATE.

Instead of the residents of East Hull having to set to work to collect funds for the establishment of a nursing home, Mr. and Mrs. F. B. Grotian have presented that portion of the town with one. The existing commodious premises of the East Hull Conservative Club in Holderness Road are to be utilised for the purpose, the club removing to the opposite side of the road. The generous donors have also taken upon themselves the obligation of fitting up the building, so that a completely furnished home will be handed over to the District Nursing Association free of all cost.

### THE LESSON OF THE ELECTRO BATH CASE.

There is a lesson to be learnt by nurses from the action which was tried last week, in the King's Bench Division, to recover damages for personal injuries received, it was alleged, by the negligence of the servants of the Medical Electro-thermic Generator Company while administering an electro bath to Mrs. Agnes Gwynne. Miss Moxon, the nurse in attendance on Mrs. Gwynne, stated that on the occasion of the first and second baths lint was used for packing purposes, but that no lint was sent for the third bath. In the absence of any, towels were employed to swathe the body, and it was held that in consequence of the substitution of the towels the plaintiff suffered injury. Nurse Moxon, in her cross-examination, declared she did not know that lint was necessary, and therefore did not inquire for it. This is in direct conflict with the statement of the company's nurse, who said that when the first and second baths were administered she herself explained to Miss Moxon the importance of the lint being placed in a certain position. The point is, however, that when a nurse has seen such a bath administered in one way, she should not, without definite medical instructions, administer it in another. The roll of lint which accompanied the bath in the previous instances should have been asked for and obtained before the third bath was given.

### ROYAL DERBY NURSING ASSOCIATION.

The thirty-sixth annual meeting of the Royal Derby and Derbyshire Nursing and Sanitary Association took place last week. According to the report of the lady superintendent, which was adopted, the staff now numbers 77 nurses, 50 of whom are engaged in private nursing, 10 in district work, the remainder being probationers in training. Last year the private nurses were employed in upwards of 500 families and 42 hospitals. On four occasions they took matron's duty. They nursed 267 medical, 75 surgical, 71 maternity, 33 mental, and 86 infectious cases, and did 2,156 weeks' duty. The district nurses and superintendent paid 36,689 visits. As to the financial position, the income was £4,074, and there was a balance of £198 to the good, which it was announced would be carried forward for special bonuses for nurses. The subscribers, the lady superintendent, and the nurses must all be congratulated upon the flourishing condition of the institution.

### GOOD ADVICE TO THE YARMOUTH GUARDIANS.

There have been upwards of a score of resignations of nurses in less than three years at the Yarmouth Workhouse Infirmary, and Captain Hervey, one of the Local Government Board Inspectors, strongly advises the Guardians to make the nurses' quarters more comfortable and attractive. In giving them this advice, he said that the demand for competent nurses was far greater than the supply, and he added it was his opinion that when once obtained good nurses should be taken great care of, well looked after, and offered every inducement to stay. Whether the Yarmouth Board of Guardians will act upon this excellent advice remains to be seen, but it is the only method by which they can hope to overcome the difficulty they experience in keeping their nurses.

### THE NURSES AT FRIEDENHEIM HOSPITAL.

At the annual meeting of Friedenheim Hospital, last week, the report of Miss F. M. Davidson, the honorary superintendent, who founded the Home of Peace for the Dying in 1885, was adopted on the motion of Mr. J. Herbert Tritton, seconded by Lord Stamford. The report stated that at the present time the sum of £10,000 is required to build a new nurses' home, for improvements and alterations in the present buildings at Hampstead, and for putting the finances on a firmer basis. At the instance of Dr. A. T. Schofield, the honorary physician, it was resolved, "That this meeting considers that the time has arrived when the erection of a suitable nurses' home has become an urgent necessity, and that a special effort be now made to raise the necessary funds for this purpose." After the nurses' home has been provided, the other portion of the scheme of improvement will doubtless be proceeded with, subject, of course, to sufficient money being forthcoming for the purpose.

### THE NURSES' UNION.

On Thursday last week an "At Home" was given at 26 George Street, Hanover Square, by Mrs. Walton, Vice-President of the Nurses' Union, in connection with the sale organised by the members in aid of foreign medical missionary work, which realised £17. After the guests, who were in uniform, outdoor or indoor, had been most hospitably entertained, several ladies and gentlemen sang and recited. In the course of the afternoon Miss Van Somner, who has just come from Sierra Leone, stated that a nurse is urgently needed there just now in the Princess Christian Hospital in that colony, where her work lies among very simple black people, who have so broken our English as to make it impossible for us to understand at first, while they understand us quite well. She also told how amusingly they express themselves as to their ailments, and said that it was often only by means of careful inquiry as to where the pain was not that the doctor could discover where it was.

### AN ANNUITY FOR A MATRON.

The friends of Miss Charlotte Rigney, who was lady superintendent of Swansea General Hospital until November, 1899, will be pleased to hear that, in recognition of valuable services rendered to some officers who were invalided home from the front in the early part of last summer, she has been left by the father of one of her former patients—an old Canadian military man, who recently died—an annuity of £80 a year. We are informed that the surgeons and nurses have also been handsomely remembered by the grateful father. Miss Rigney has no intention of retiring, but is taking up another branch of nursing work.

### MORE ARRIVALS FROM SOUTH AFRICA.

The following sisters have arrived in charge of sick and wounded from South Africa:—On board the ss. *Assaye*: Sisters Campbell, Goldsmith, Morris, Ahnett, and Horne; Sister M. J. Spencer (A.N.S.R.) was invalided to Netley. On board the H.S. *Orcana*: Sisters A. C. Jacobs, H. G. Young, W. Newton, A. G. Cocks, and J. Campbell. Miss Newton is a member of the New South Wales Army Nursing Reserve, and is in England for the first time. Miss Cocks is from South Australia, and Miss J. Campbell from New Zealand. The *Orcana* brought some very serious medical cases, including convalescent malaria, and one case of very serious wounds.

### A NURSES' HOSTEL AT MANCHESTER.

A Manchester nurse informs us that the nurses' hostel in that city is now in full working order. "We have two pleasant sitting-rooms, one in the front, gay with flowers, books, and plants, and rich in real easy chairs and a deliciously comfortable couch; the other in the back is quieter—some would say duller—and cooler, being away from the afternoon sun and the road. There we have our meals daintily served with nice linen, bright glass, silver, and china, and also read and write quietly. The bedrooms are very comfortable, and the matron does her best to make us feel at home. It is taken for granted that a nurse works hard when she is at a case, and therefore needs and deserves a good rest betweenwhiles, so breakfast is served to us in bed as a matter of course and without any extra charge. The hostel is conveniently situated in a good broad street between two of the principal tram routes, near to many of the leading Manchester doctors, and close to shops and other conveniences."

### SHORT ITEMS.

On Monday afternoon a meeting was held at Londonderry House with the view of forming a ladies' committee to organise in the City of London the Women's Memorial to Queen Victoria. The objects were briefly explained by the Marchioness of Londonderry and the Duchess of Portland (two members of the executive committee). A representative committee for the City was formed, under the presidency of Lady Dimsdale, with power to add to their number.—The sum of £227 1s. 4d. has been received by the Maternity Charity and District Nurses' Home, Plaistow as the result of the recent café chantant at Lord Brassey's house in Park Lane.—Miss Stobart, who has for many years been district nurse at Ryton-on-Tyne, was married in the old parish church on Wednesday, June 5, to Mr. Andrew Gemmel, of Newcastle. Many handsome presents were received, including one from the District Nursing Committee.—On the invitation of Miss Seton-Karr, a garden party will be given at Hermon, Lingfield Road, Wimbledon, on Tuesday next, for the purpose of explaining the objects of a Nurses' Volunteer Missionary Union. Tea and coffee will be provided, and addresses will be given at intervals between three and seven. Army nurses who can attend will be welcomed; but they are asked, if possible, to signify their intention of being present to Miss M. Cable, Gordon Hall, Gordon Square, W.C.—The Clapton Surgical and Medical Home in Southwold Road, Upper Clapton, has been considerably enlarged and now accommodates from 18 to 20 patients, while the staff for outside work comprises 20 to 30 fully-trained nurses. One of the new features is a small operating theatre, and one of the wards is fitted for Weir-Mitchell cases.

### Notes of Surgical Lectures to Nurses.

Delivered at the London Temperance Hospital by Dr. W. J. COLLINS, M.S., B.Sc., D.P.H.Lond., Fellow of London University and of the Royal College of Surgeons. Surgeon to the London Temperance and the Royal Eye Hospitals.

### ABDOMEN—DIGESTION.

UNLIKE the thorax, the cavity of the abdomen is only partially walled in by bones. The diaphragm, supplied by the phrenic nerves, forms its mobile roof; below it is continuous with the bony pelvis made up of the two innominate bones, the sacrum and the coccyx. The five lumbar vertebræ form its posterior wall, while muscles make up its lateral and anterior parietes. The linea alba, from the xiphoid to the symphysis pubis, marks the union of the two symmetrical halves, and is sometimes the site (especially at the umbilicus) of hernia or rupture. Three transverse lines, one at, and two above, the umbilicus, show the tendinous intersections in the recti muscles. Separating the abdomen from the thighs are the two ligaments of Poupart, which stretch from the anterior superior spines of the iliac bones to the pubes: above and below the inner extremities of these ligaments inguinal and femoral herniæ are apt to occur. If two horizontal lines are drawn around the abdomen, one at the level of the ninth costal cartilages and the other at the level of the anterior superior iliac spines, and two vertical ones drawn upwards from the middle of Poupart's ligaments to the eighth costal cartilages, the abdomen is mapped out into nine regions, which serve to localise the viscera in health and the presence of tumours, &c., in disease. They are from left to right and from above downwards: — (1) Right hypochondriac; (2) epigastric; (3) left hypochondriac; (4) right lumbar; (5) umbilical; (6) left lumbar; (7) right iliac; (8) hypogastric; (9) left iliac.

The liver, the largest gland in the body, weighs 50 oz., and can be felt just below the right costal cartilages in the right hypochondriac and epigastric regions. It secretes bile, which is stored up in the gall-bladder, and elaborates from the products of digestion a substance intermediate between starch and sugar called glycogen, and stores it up for future use. The liver, like the other organs in the abdomen, is covered more or less with a delicate layer of serous membrane called the peritoneum, which also lines the walls of the abdomen and suspends the intestines by its folds, called mesenteries. When this membrane is inflamed (peritonitis) there is fever, great abdominal tenderness, and fluid may be poured out into the peritoneal cavity. The stomach, continuous at its cardiac end with the œsophagus and at the other (pylorus) with the commencement of the small intestine (duodenum), lies in the left hypochondriac and epigastric regions under cover of the left costal cartilages and the left lobe of the liver; its capacity when distended is about four pints: on its left is the spleen. The coils of small intestine (20 feet), consisting of duodenum (8 inches), jejunum (about 8 feet), and ileum (about 12 feet), lie chiefly in the umbilical and hypogastric regions and terminate in the right iliac region (ileo-cæcal valve) in the commencement (cæcum) of the large intestine (colon) which passes up to the liver, across the abdomen below the stomach, and down the left lumbar region to terminate in the sigmoid flexure and rectum. The large intestine is five feet long: it is larger in bore and more fixed than the small intestine and is sacculated; attached to its blind commencement is a vermiform appendix which, by reason of the irritation of foreign bodies, concretions, or inflammation, may cause peri-typhlitis or appendicitis which may require operation. The pancreas lies deeply behind the stomach, stretching across the abdomen from the spleen to the duodenum into which it pours its secretion by means of a duct which enters the gut along with, or close to, the bile duct which proceeds from the liver and gall-bladder. The aorta enters the abdomen through a hole in the diaphragm and bifurcates at the left of the fourth lumbar vertebra into the common iliac arteries, which after further division reappear in the groins as the femoral arteries. The inferior vena cava in a corresponding fashion commences as a common trunk on the right side of the fifth lumbar vertebra, grooves the under surface of the liver where it receives the hepatic veins, and passing through the tendinous centre of the diaphragm arrives within the pericardium and pours its blood into the right auricle. The returning blood from the stomach, intestines, and spleen is collected by the portal vein which, laden with the products of digestion passes to the liver and again into capillaries to be finally re-collected in the hepatic veins and vena cava.

Digestion is the process whereby materials from the outside world in the shape of food are so dealt with as to subserve the purposes of repairing the tissues and cells of the body and of evolving energy (heat, motion). Just as the body, viewed microscopically, is said to consist of cells, so the cells may be said chemically to consist of protoplasm. Protoplasm consists of water, salts, albumin or proteid, carbo-hydrates or starchy matter, and hydro-carbons or fat: reduced to elements it consists of carbon, oxygen, hydrogen, nitrogen, with smaller quantities of sulphur, phosphorus, potassium, sodium, and chlorine. Food is the source whence, by the intermediary of the blood, cells derive their sustenance and energy: it must therefore be correlated to the composition of protoplasm. Digestion, like respiration, is external and internal: external digestion takes place when foodstuffs are submitted to the action of digestive juices on the walls of the stomach and intestines; internal digestion takes place when the blood, charged with the products of digestion, supplies pabulum to the cells of the body in the systemic capillaries. Just as the corpuscular part of the blood is mainly respiratory in its function, so the plasmic part of the blood is mainly digestive in its destiny.

Milk is an ideal food, and contains samples of all the needful components of a normal dietary; besides water and salts milk contains proteid (5 per cent.), fat (4 per cent.), sugar (4 per cent.). A normal dietary for an adult should consist of about 3½ ozs. of proteid, 3½ ozs. of fat, 8 ozs. of carbo-hydrates (all water free), 80 ozs. of water, and 1 oz. of salts.

By the action of the saliva, the gastric juice, the pancreatic juice, and the bile, the meat, bread, butter and potatoes, &c., of a meal are reduced to a state of solution or emulsion, and so rendered capable of absorption into the blood vessels or lymphatics (lacteals). The starchy matters (carbo-hydrates) are reduced to grape-sugar or dextrose by the action of the saliva (ptyalin) and pancreatic juice (amylopsin). The nitrogenous or proteid bodies are reduced to the form of peptone by the acid (HC1), gastric juice (pepsin), and alkaline pancreatic juice (trypsin). The fats are saponified and converted into fatty acids and glycerine by the pancreatic juice (steapsin) and emulsified and rendered capable of absorption by the bile. Water, crystalline bodies, and soluble salts are absorbed without change. Thus the net products of digestion are sugar, peptone, water, salts, and saponified and emulsified fat.

### To Nurses.

**WE invite contributions from any of our readers, and shall be glad to pay for "Notes on News from the Nursing World," or for articles describing nursing experiences, or dealing with any nursing question from an original point of view. The minimum payment for contributions is 5s., but we welcome interesting contributions of a column, or a page, in length. It may be added that notices of entertainments, presentations, and deaths are not paid for, but, of course, we are always glad to receive them. All rejected manuscripts are returned in due course, and all payments for manuscripts used are made as early as possible after the beginning of each quarter.**

### Suggested Reforms in Military Nursing.

By an Army Sister in South Africa.

The Hospital Commission has come and gone. Round it centred, perhaps unreasonably, the hopes of many that the reform of the military nursing system was close at hand, though there were not wanting pessimists, let us not say prophets, who insisted that after every great campaign there had been Commissions, that their deliberations bore no fruit, and that things would be exactly the same in the next war.

If the scope of the Commission does not embrace, as its published report would lead one to suppose, nursing reform proper, it has served at least to draw widespread attention to military hospital methods, and as the flood of knowledge and light pours on them, better conceptions of what is possible for and due to sick soldiers, more enlightened thoughts as to the requirements of modern surgery and medicine, broader and higher ideals of management and organisation will necessarily result.

The Report breathes such a spirit of tolerance and large-mindedness, while yet conveying to the initiated a very clear picture of the shortcomings, abuses, and tragic deficiencies of the hospitals under the observation of the Commissioners, that military authorities should give due weight to all the recommendations it submits. Even those which barely touch the subject of "nursing," if carried out honestly in their true spirit, would pave the way to many reforms which would closely affect the well-being of our sick soldiers. Take, for example, the recommendation that military doctors should have leave to study at some of the large hospitals from time to time, thus keeping pace with the strides of medical science, instead of finding themselves, through no fault of their own, behind their civilian confrères. And again, take the Hospital Commission's recommendation that there should be some reasonable limit to the demands made on the time of senior medical officers by purely secretarial work, giving them greater leisure to attend to organisation and the real business of hospital administration; this amplified, would include a radical alteration in the system of dieting patients. The present army practice is embodied in a large sheet of blue paper, ruled with spaces for every day of the month (a fresh paper having to be issued from the wardmaster's office and filled in for each month). On this paper every item of diet, not included under the headings "Light" or "Rations," for each patient has to be written down by the medical man every day and initialed. For instance, if a patient is on porridge or mince or two eggs in addition to his milk diet for every day in May, each one of those items has to be written down thirty-one times, and when perhaps there are six extras to be chronicled and thirty patients to be dieted (to take a very small military ward as example) some idea of the tremendous waste of the doctor's time and energy can be gained; and time thus spent has to be subtracted from what should be real medical attendance on the patients, from a full observation of symptoms and the making of careful diagnoses.

### Nursing at Military Hospitals Abroad.

While the Hospital Commission has in these and other ways helped to clear the ground for military nursing reform, it would be well that suggestions and criticism should come from within—that sisters, military and civilian, who have worked under the army system, have appreciated its good points, have suffered from its unreasonableness and its hard and fast limitations, should have opportunity to express their opinions and experiences; and that out of the mass of fact and knowledge some workable reforms should be introduced into what might be a thoroughly up-to-date service instead of a high-water mark of nursing ideals 20 years ago. Criticism made from honest and disinterested motives should at least be received with forbearance and need not involve bitter recrimination. Many sisters must be able to speak from knowledge of numerous military hospitals at home and abroad. Even under military routine, different institutions emphasize various characteristics of the system, and speaking from my own experience of three, I would unhesitatingly state that the standard abroad is far higher than at the large home station where my lot at first threw me. Less red tape, larger ideas of what may without loss of dignity be done by sisters for soldiers, more discipline, and better behaviour among the orderlies—all of these are more accentuated abroad. Of course much depends on the personnel of the medical staff, and something on the sister superintendent, but, speaking broadly, I think many will corroborate the above statements. That this within its present restricted limits has been accomplished in new surroundings, *i.e.* South Africa, is a sort of pledge of the possibilities of the system when re-organised and set free from the trammels of old customs.

### Reforms—The Orderly Question.

What reforms are necessary in military nursing?

Many and various replies will be forthcoming to such a query, reflecting the temperament, judgment, and experience of the individual, but there are a few broad headings under which many details may be grouped; some direct lines along which military nursing must move if the goal of reform is ever to be reached. And first and foremost I would put the "Orderly" question; it is the crux of the whole matter. Not the abolition of orderlies—an impracticable project so long as the possibility of war has to be reckoned with—but some plan which will raise the standard of conduct, professional skill, and general intelligence among a body of men where conscientiousness and knowledge are essentials to the proper discharge of duty. In all civil hospitals a period of probation, from 1 to 4 months usually, before acceptance for the nursing profession is compulsory. Time is given that the candidate's aptitude for learning details, moral character, and mental capacity may reveal them selves, and judged by this tentative experience she is chosen or refused as probationer. Nothing of this kind finds a place in the R.A.M.C. as far as orderlies are concerned—once an orderly always an orderly. There is no kind of selection—a man enlists in the corps presumably for the higher pay that he receives than he would as fighting Tommy Atkins, and very probably thinking that he also secures an easier billet than the ordinary privates; his nursing enthusiasm survives for a week or a fortnight, then, rudely awakened to the fact that nursing means plenty of hard work, he settles down to the daily routine, content to do, in his own fashion, what he is told, but no more. Nothing short of some really heinous offence (not drunkenness) will suffice to rid the service of an utterly incapable, unsuitable man. He must be endured by patients and sisters, because such reasons are not sufficient to discharge him from the R.A.M.C. I well remember two orderlies at different hospitals to whom all that has been said strictly applies. No. 1 was reported by every sister in the institution, as thoroughly unreliable, as eating the patients' food, as being asleep over and over again on duty, as being a confirmed prevaricator of the truth, neglectful, and incompetent. Yet that boy, though put to do scavenging work as the only suitable employment for him, is still a member of the R.A.M.C. and more than likely is now in charge of enteric cases. No. 2 would have adorned a stable or found a vocation as coal carrier. He was reported by the soldiers as resembling a young elephant in the ward, clumsy, noisy, and utterly oblivious that patients were sick people; yet, after repeated complaints from the sister, he was still responsible for enteric patients. Though these are glaring instances I venture to think that any sister could select from any military hospital two out of every ten orderlies who are unsuited in one way or another to their work.

*(To be continued.)*

### The Ilkley Hospital and Convalescent Home.

By the Matron.

The Ilkley Hospital owes its origin to the Rev. George Fenton, who in 1829 was curate-in-charge of the parish.

It has been known by various titles, the sum-total of which is eighteen, and the present name was curiously come by. In the early part of 1886 a legacy of £1,000 had been left to "The Ilkley Convalescent Home," and had been paid to the Bradford Corporation as custodians of the Semon Convalescent Home at Ilkley; eventually the sum was handed over to this institution, but it was deemed advisable to supplement the title by the words "and convalescent home."

It is a fine building, standing in its own grounds of three and a third acres, where the patients are allowed freedom to roam about as they like. Many seats are provided in various parts of the gardens and enclosures for patients unable to climb on to the moors. The home contains 100 beds; fifty-six of these are for females, and the remaining forty-four for males.

### The Patients.

Separate bedroom accommodation is provided for the use of phthisical patients in an early stage of the disease. So far as is possible these patients live an out-of-door life; special care and attention is given to prevent others from falling victims to the disease. Sputum bottles are given to each on his or her arrival, with printed regulations for their guidance in sanitary precautions. Cases of rheumatism are found to derive very great benefit from the Ilkley Baths and moor air. These baths are situated on the edge of the moor, and it is the water of these "Old White Wells" which has made Ilkley famous. Here our patients get douche, shower, and plunge baths free if medically ordered. On entering the hospital every patient is weighed before being seen by the medical officer, when the usual details are taken of their position and disease (if any), and a certain grade of work is entered in the official record and given to the matron to apportion to each individually.

### The System of Work.

Great care is taken to dole out the work so that there shall be as little grumbling on this score as possible. Each week the work done by each patient is altered so that during the usual stay of three weeks three different classes of work is done by each able-bodied inmate. Any crippled by rheumatism or otherwise are given the knives and forks to clean, or potatoes to peel, or some other job at which they can work sitting down. Six patients are usually told off to set the tables, another six to clear them, and so on; men and women working happily and sensibly together. Should an undesirable patient get admitted by some mischance, the matron has the right to discharge him or her. In this way discipline is kept up. It is quite remarkable how little goes wrong with these patients when the rules are so few, and so little supervision given them. About one dismissal a year covers the number, and the effect is felt long afterwards. No redress being given, the offenders are those who suffer.

### The Diet.

The diet is most liberal; testimony to this fact is borne by the extra weight in which most of them rejoice in at the end of their visit. The meal times are as follow:—Breakfast, consisting of bacon, eggs, or fish, with tea or cocoa, at 8.15 A.M.; dinner of mutton, beef or fish, with pudding every day 1 P.M.; tea at 5 P.M. of bread and butter and sweets; supper at 8 P.M. of porridge and milk, bread and milk, or milk and bread and butter. Patients are allowed to provide themselves with extra luxuries, but the above are regularly and liberally provided. It is good to hear the hearty way in which these North-country people sing Grace, before and after food. They seem to sing as though they really are thankful for their food in plenty. The tables are supplied with plants and flowers when possible, and it is seldom they are without. Visitors are allowed to see the patients every day, but Wednesday and Saturday are the favourite days, as cheap trains run from Leeds, Bradford, Harrogate and York. During the summer two carriages are supplied every week for drives for those patients who are unable to get the moor air by walking over it. These drives are given by two ladies living in the neighbourhood, and they are a real pleasure and treat to those who are selected by the matron to go.

### The Nursing Staff.

Both myself and the sister are trained hospital nurses so that those who require extra care and attention are able to have everything which their case requires. In this way, too, there is little chance of patients being given work to do for which they are physically unfit. Patients are admitted into the home by letters of recommendation through a subscriber. These letters are equivalent to a guinea, and allow of a three weeks' visit. Patients wishing to stay longer are able to do so by payment of 7s. a week or by procuring another recommendation, if the medical officer considers the case as a suitable one, and likely to benefit by the longer stay, and as such recommend it to the managing committee. Seven female servants form the kitchen staff of this hospital. They constantly clean and scrub the hospital; do the laundry work; and one of them makes and bakes the bread consumed in the place. All work happily and unitedly together. They are of various religious denominations, like the patients themselves. No exception is taken to either from point of religious differences, and I think that we can boast of being a united band of workers.

### The Presentation of War Medals.

By a Disappointed Spectator.

At an early hour on Wednesday morning, I started out to go to the Horse Guards Parade, to see the presentation of war medals by the King. The sky at first looked threatening, and as I neared Whitehall a slight shower fell; but it was quickly over, and I was glad that I had made up my mind to stand in the crowd, so that I might tell the nurses who read the Mirror how the recipients of the medals looked, and how the crowd cheered, and how proud we all felt that the nursing sisters ranked with officers and chaplains in the honours gained in war. I could see in my mind's eye the little company of nurses in scarlet capes, among the brilliant uniforms of the soldiers and foreign representatives and the bright green of the trees, and I was meditating on all this, when, in a moment my hopes were dashed to the ground. I bought a paper and this is what I read under the heading, "The Honours of the War: Medals at the Hands of the King":—"The nurses, who had been included in the prepared list of recipients, will now be omitted from the proceedings, and will, it is understood, receive their medals at a later date." Alas! for my hopes. The sun shone and the crowds cheered; and the King, in full uniform with an escort of the Life Guards, and the Queen and one of the Princesses, both in deepest black, drove by. But I could not feel more than a chastened interest in the proceedings, for the "Ladies of the Army Nursing Staff" figured only in the "official" programme, and I could not help feeling disappointed. "Women always come in second," said a nurse who was standing by.

### Royal British Nurses' Association.

### ANNUAL MEETING.

THE Imperial Institute was again this year the scene of the R.B.N. Association's annual meeting, which took place on Thursday afternoon, when a very considerable audience of members and friends assembled to receive the annual report and balance-sheet. Sir JAMES CRICHTON-BROWNE presided, and he was supported by Mr. Fardon, Mr. Langton, Sir Charles Gage-Browne, Miss Thorold, and Mrs. Dacre Craven. A preliminary meeting had already taken place, at 10 Orchard Street, of Lady Consuls of the Association and matrons on the General Council. The Treasurer's report was taken first.

Mr. LANGTON said there was abundant evidence of vigorous growth of the Association and good promise of a still larger development. He would like to emphasise the usefulness of the Helena Benevolent Fund: one nurse was receiving a pension of £20, and two others in confirmed ill-health had received weekly allowances during the year to enable them to live in more comfort. Many grants had been made to members in convalescence and in temporary distress. It was, perhaps, rather a hard thing for a treasurer to say, but he thought they ought not to begin to build the settlement until they had at least £3,000 in hand.

### THE ANNUAL REPORT.

Mr. FARDON moved the adoption of the annual report, which showed an increase of members to the number of 169. One hundred and seventy-four nurses were accepted by the Registration Board, but a larger proportion than usual were rejected on account of insufficient training or for other reasons. The South Australian branch had been successfully organised under the vice-presidency of Lady Tennyson. Miss Graham, matron of Adelaide Hospital, was Lady Consul for the whole district. Miss Dorothea Pizey was hon. secretary and treasurer. A large number of new members were ready for registration and enrolment, and the prospects of the branch were very encouraging. The Auxiliary Nurses' Society, founded at the beginning of the year, was already doing good work, and there appeared to be no doubt that a large field of employment was open to the nurses who had joined it. Mr. Fardon said he thought they might now, after fourteen years, pause and look round. The Association might very fairly claim to have laid the foundation, and to have made some progress in all the purposes set forth in the Charter. There had been very few instances in which they had had to exercise the prerogative of discipline. The bureau was by no means a small part of the work, and the result was that they were able to show that during last year over £14,000 had been earned by nurses of the corporation. He believed that they might look forward to extension and advance. Not so many nurses had, however, joined the Association as he thought should have done. There were approximately 30,000 trained nurses in the country; the Association numbered 3,000. The proportion ought to be one-half instead of one-tenth. With even 10,000 members, it would not be difficult to organise some scheme by which each one might look forward to a comfortable old age.

### THE SETTLEMENT SCHEME.

The Association, Mr. Fardon continued, had no desire to interfere with any other such scheme, such as the Royal National Pension Fund, with which they were in cordial sympathy; but he wished specially to allude to the settlement scheme, which had been so auspiciously started and so efficiently helped. The Association was by far the best organisation of the kind: it was under Royal patronage, and it numbered among its members many ladies who were or had been in the past matrons of hospitals. They had had to do with the training of nurses, and were able to influence any who came to them for counsel and advice. But they ought to have the assistance of more matrons. He was afraid that some were holding back, not because they were not convinced of the utility of the Association, but because they did not like the public criticism to which it was possible they might be submitted. But if they waited until all men spoke well of them, they would have to wait some time. What institution was not subjected to attack? Adverse criticism did not do the slightest harm, and the Association ought not to suffer from it. Another expression of opinion was that, according to the legitimate purpose of the Association, as laid down in the Charter, members ought not to mix themselves up with any political matter. The public estimation in which nurses were held depended on how people saw they did their work; to the extent to which they did this, and refrained from mixing themselves up with public matters, so far would the Association continue to enjoy public support and to do good work.

Miss SCOTT, in seconding, read a short report on the work of the Lady Consuls; it had been found that the Settlement scheme had always met with approval from those nurses with whom the Lady Consuls had come in contact.

The report was unanimously adopted.

Sir CHARLES GAGE-BROWNE spoke of the intense sympathy and interest always shown by their President, H.R.H. Princess Christian, who was unavoidably absent. She had steered them through many difficulties, and he remembered how she had remained faithful to them even when things went so far that it was hardly consistent for a gentleman or lady to remain in the position she held. He proposed that a very sincere vote of thanks be sent from the meeting, and reminded those present of the losses their President had sustained, and that a resolution of condolence had been sent by the Association at the time.

### ADVERSE CRITICISM STIMULATING.

Mrs. DACRE CRAVEN said that she as well as Princess Christian had sent a son to the war, and the fact that he still lived had made it terribly difficult to know how to write to their President in her great sorrow. With regard to adverse criticism, she thought a little of it a very good thing; an Association, like the Government, was better for an opposition; she had once been hon. secretary when a writ had been served on her. Nurses had a great deal of power, if they would try and think for others; they had to think for their patients as for children. She would like to give them a very cordial invitation to the unveiling of a statue, outside St. Andrew's Church, Hoxton, on Wednesday, the 19th, at 4 o'clock. It was put there by the Cordwainers' Company, in memory of the doctor to whom was due the foundation of the Royal Free Hospital and the Cancer Hospital. He had found a poor girl dying on the steps; in those days hospital accommodation was very much smaller than it was now, and not one of the hospitals would take her in. They all knew how sympathetic doctors were; he had taken a lodging for the girl, where he attended her till she died.

### "PSEUDO-ANGELS."

Sir JAMES CRICHTON-BROWNE said he did not admit that opposition was necessary. There had been no sudden rush, but a steady progression all along the line. He was afraid the public did not yet realise the advantages of efficiency and some kind of discipline. Years ago doctors had found it necessary to establish a Medical Council, and so now in the nursing profession some central control was necessary for ensuring proper qualifications, and for eliminating those who were inadequately trained. A lady had recently told him that she was not surprised that insanity was increasing, seeing that there were so many doctors and nurses about; she had just been brought to the verge of insanity by having two "pseudo-angels" in the house. He had recommended her to apply to a certain society which he would not name, and she had since withdrawn her statement, at any rate, so far as the nurses were concerned, for she had found the fresh nurses better and brighter even than the angels Coventry Patmore had described. The object of the Association was to get rid of pseudo-angels.

After the meeting was over the nurses went to the Crystal Palace for their annual excursion, reserved first-class carriages being kindly arranged for by the railway company. Through the courtesy of Messrs. Lyons, who placed one of their rooms at the disposal of the Association, an excellent view of the fireworks was obtained, and the same firm catered for the tea.

### Nursing Plague and Enteric: Experiences of a Colonial Nurse.

BY A CORRESPONDENT.

MISS FRONEMAN had not been in England twenty-four hours when I called on her, and she was still so tired with the effects of the nursing during the voyage from the Cape, that it seemed almost cruel to ask her to tell me about her work in South Africa. Nurses, however, when they love their profession, are always ready to set aside their own feelings, and Miss Froneman assured me that she would gladly give any information which might be of use or interest to readers of "THE HOSPITAL" NURSING MIRROR.

### PLAGUE PATIENTS.

"I was one of four," she said, "who volunteered, some time ago, to nurse the plague if occasion required. Most people are afraid of the infection, and Colonials have as a general rule a horror of plague. It is so closely connected with dirt; and English people have no conception of the state of filth in which the Cape negroes live. The blacks are the cause of this outbreak."

"But it has attacked the European population, has it not?" I inquired.

"Yes, and unfortunately Europeans rarely recover from it; their constitutions have not the power to resist it. Look at poor Miss Kayser, and her own sister who nursed her; they died within a very short time of one another. It was terribly sad."

"You do not fear the infection?"

"No. I have been inoculated for it twice, and I have no fear. I never touch a patient without washing my hands in carbolic, and I take a carbolic bath every night."

"Is it likely that the cold weather will lessen the chances of its spreading?"

"Dry cold would, but not cold foggy weather. The plague germ lives in mud, I was told by the plague specialist, and as long as there is any damp it is not likely to abate. The accounts are, however, better since I left Capetown; then we had thirteen cases a day, and sometimes fifteen; eleven in a week is to-day's report, and that is a great drop. It is the pneumonic form of plague, from which recovery is always very doubtful. My patients were all coloured people."

"Is there any law in Capetown to prevent the mixing of the white and coloured population?"

"No; there are no police apart from the military, and the finest lady or gentleman may sit next to the filthiest black in a tram. They may even live next door to a Malay family; the servants mix, and when habits are dirty and sanitation imperfect, it is not surprising that the disease spreads. In Johannesburg there are the strictest regulations; trams and trains are marked 'Coloured'; it is quite necessary, and it would be a very good thing if the same were done in Capetown. It is not Zulus or Kaffirs to whom I refer, but Cape negroes, who are much lower in the social scale, but who have a great idea of their own importance."

### AT THE SOMERSET HOSPITAL.

"You were trained in South Africa, were you not?"

"Yes; I have been for seven years at the Somerset Hospital, where I am now Theatre Sister. But I am known as Nurse Hettie, because the matron being an All Saints' sister, the title would be likely to get wrongly used if we were all called "sister." I have also very responsible work in training the probationers. We have both English and colonial nurses."

"Have you heard many complaints from colonial nurses against the War Office for not employing them for military nursing?"

"Yes; and my own opinion is that colonial work should be given to colonial-trained nurses. The greatest difficulty English nurses have is in the heat; many of them have told me repeatedly that they simply cannot stand the heat. You in England don't know what heat is; you think it is hot to-day, and I have been wondering what it is like when it is really cold!"

### WANTED: A MILLIONAIRE.

"But it is a healthy climate, on the whole?"

"Oh, yes; the cold dry season is delightful; there is a broiling sun up to 4 o'clock in the afternoon, and then cold invigorating breezes. It is the very climate for phthisis, only I wish patients would come out before they get to so advanced a stage that nothing can save them."

"It is said that the Karoo would be a splendid place for a sanatorium."

"Doubtless it would; but it wants a millionaire to build it. I should like very much to see it done; there ought to be two houses, one for poor people and the other for those who can afford to pay, with a matron and a proper nursing staff; it ought to be a tremendous boon. I wish Mr. Rhodes would do it."

### NURSING UNDER DIFFICULTIES.

"This is your first visit to England, I think?"

"Yes, and I am so glad I came. I had heard so much about nursing on the transports, and at last I thought I would try it. We brought 1,600 men over in the s.s. *Avondale Castle*, and it was a very good thing there were sisters on board, for though the ship started with a clean bill of health, at least thirteen very serious cases of enteric developed after we had left the Cape. And there were other illnesses—intermittent fever, dysentery, jaundice, in fact all sorts of gastric complaints. The officer said when we were leaving, "You wont have much to do, Sister, for there's no one sick." The men had been sent straight down from the front, on to the ship, without being allowed a minute in Capetown; the train simply ran them into the docks, and they were off before they knew where they were, so as to avoid the slightest infection from plague; but they must have had enteric in them, for it very soon began to appear. The heat was fearful, and I shall never forget how we worked; there was never a morning that we were not down in the hospital by five o'clock, sponging and giving ice-packs. Imagine giving a sponge bath standing on a box and reaching up to the top bunk, in danger of falling over backwards from the rolling of the ship."

"How many sisters were on duty?"

"Only two, Miss Horder and myself. We were never on deck till half-past twelve; it took us till breakfast time to attend to the men, give medicines and stimulants, &c., and after breakfast we went to the third-class saloon, where we attended to the less serious cases, gave medicines, and dressed sores. This saloon was over the screw, and the vibration was dreadful. All the really bad cases came to us, and the rest were under the care of the orderlies."

"Did you lose any patients?"

"Yes, two poor boys died; such nice fellows. We could not keep the temperatures down; one was 106 more than once. I was so sorry for one poor fellow, for we were coaling at Las Palmas, and the coal was being shot down very near where he was lying. He fought so hard for life, in that distressing noise."

"Of course you did not go on shore?"

"No; apart from being under the yellow flag, we were much too busy to leave our patients."

Miss Froneman, who is a member of the Royal British Nurses' Association, has six weeks' leave, and she had made up her mind to see as much as possible of London in that time. She specially wants to see some of the slums of which she has often heard. She was particularly struck, she said, with the wonderful order of the streets in the City, and the almost superhuman power of the policeman's hand. The lovely scenery of the New Forest had, of course, appealed to Colonial eyes, as the train travelled up to London from Southampton on Sunday evening. "We have nothing like your green hedges in South Africa," she said.

### Nursing in the Malay Settlements.

By an English Nurse.

Nurses who go abroad are often sadly disappointed because there are so many difficulties to contend with, and it may perhaps be some comfort to them to know that we in the Federated Malay States have not found nursing "couleur de rose" either.

It is important that all who intend working abroad should understand the work, the difficulties, the disadvantages, the salary a nurse receives, the expenses of living, the people she works for, and the general absolute lack of interest displayed in nurses and their work generally, even by those connected with nursing committees.

In the belief, therefore, that it is part of the duties of the experienced to inform others of their experience, I have taken it upon myself to submit to my professional sisters the following brief sketch of the vicissitudes and drawbacks attending the introduction of trained nursing to a little Eastern State.

### The Pioneer Nurse.

My predecessor was placed in charge of the General Hospital, containing about 80 beds, and a ward set apart for Europeans with four beds. Her difficulties were great and the work was arduous. Besides nursing the Europeans, who were entirely dependent on her services, she had to superintend the native wards, containing practically nothing but Chinese and Tamil coolies, male and female. After a short attempt, owing to friction, which might have been anticipated, between the Eurasian apothecary, his staff of dressers and the nurse, she was obliged to protest, and subsequently was placed partly in charge of the European ward. Here she worked for ten months, then got married, repaid her passage money, and went home.

### Our Quarters.

The arrival of my colleague and self in this State was far from encouraging. We found ourselves in a totally strange place, knowing nothing of the language, and there was not a soul to meet us or direct us anywhere. The town, though in some ways very primitive, luckily boasted of a hotel, whither we two friendless females eventually betook ourselves. But it was not easy to get there, as the ricksha coolie understands no known language, and can only be directed bv sundry pokes to right or left. The next morning the secretary of the Nursing Association and a friend bethought themselves of our existence, and paid us a visit. But it may be imagined that we smarted under the neglect shown on our arrival, in spite of the friendliness of our visitors. No offers of hospitality were made, and we were directed to our quarters. These consisted of half a semi-detached Tamil dressers' quarters next the lunatic asylum and the General Hospital, containing two small rooms—a bedroom, which we were obliged to share and a sitting-room. The furniture consisted of two beds, a few chairs and a packing-case for a dining-table—the table was supplied later. There were no provisions, no kitchen utensils, and no servants engaged. The doors were so ill-fitting as to make shutting up at night an impossibility; there were no locks, and the doors were tied up with string. There was no verandah, and the quarters have since been occupied by a native dresser. The other half of the "mansion" was unoccupied, and after living for a little while in extreme discomfort we asked that it might be also given us. This was peremptorily refused on the grounds that the quarters assigned to us were quite good enough, and that we had nothing to complain of.

### Cooped up for Seven Months.

People at home cannot possibly imagine what it meant to be cooped up in one small room at a moist shade temperature of 87° to 90° in the Tropics, journeying to and fro in a moist sun temperature of 140°. This we endured for seven months, then both of us tendered our resignations, upon which the resident's wife, who had recently returned from home, interested herself on our behalf, and obtained for us a spacious bungalow close to the European ward, which we now occupy.

The former quarters were five minutes' walk from the European ward, along a road absolutely devoid of shade. Being in sole charge of the European ward, the nurse had sometimes to spend the night in the ward, and then tramp back in the morning to her home.

### The Colonial Secretary Intervenes.

Some improvement has taken place within the past two months owing to the intervention some time ago of the Secretary of State for the Colonies, who ordered that Government nurses should be supplied with fully-furnished quarters. I was allowed to make my own list, and have since been supplied with almost everything that is really necessary to a household; so that less expense will be entailed by a nurse arriving in the States in future than I experienced on my arrival.

### The Work.

With regard to the work, the most unsatisfactory and trying point about it is its irregularity. The European ward here has been empty for a fortnight at a time on two or three occasions, and once, when under repair, for three months. At one period there may be no severe cases, at another all may require careful nursing. But when grave cases come in it is quite a common thing to be on duty for 22 hours at a stretch, and then again the next day for 19 hours or so. My own average during the past three months works out at 17 hours a day duty, that is absolutely in charge of the cases. And, indeed, one cannot consider the two or four hours which one gets off daily as being in reality off duty. The native dressers who take one's place have so frequently proved themselves ignorant and useless that no case is ever off one's mind until convalescent and about to leave hospital. All the ordinary duties, such as indents, for diets, superintendence of same, washing patients, &c., are part of the daily routine. Special foods must be made personally, as the Chinese cooks are not capable of any cooking of an invalid kind.

### The Salary and Expenses.

The monthly salary of a Government matron in charge of the European hospital here is similar nominally to that offered to matrons with regular hours and regular leave periods at the majority of small hospitals in England. But in point of fact this appointment is only on a par with ordinary charge nurse appointments at home, as there are no perquisites, such as food, fuel, lights, servants, and laundry. The salary is $133 (£13) a month. This includes everything, even uniform allowance $8 (16s.) a month. The expenses are:—wages, market expenses, stores, oil, fuel, milk, &c., and drinks. With regard to wages, one is obliged in the East to keep a staff of at least four servants, consisting of a cook, a boy or house-servant, a water-carrier or bath-room man, and a gardener, as each one does his own particular work. The gardener may seem a superfluous expense, but he is really a necessity, for, unless your compound is kept in order, the Public Works Department interfere. Therefore, the list reads:—Cook, $12; boy, $12; water-carrier, $9; gardener, $10; dhobie, or washerman, $8; total, $51. If two nurses happen to be living together, as in our case, the total amount is reduced by half. Market expenses, which I have carefully worked out from a well-kept market-book, average $60 a month, stores, *i.e.*, tea, coffee, sugar, butter, salt, &c., $20, firewood (no coal), oil, and milk $13. Drinks average $20—showing a total of $164 a month for two nurses, or $84 a month each.

STIMULANTS OCCASIONALLY NECESSARY.

As a matter of fact when the Association nurse is at a case and I remain alone, my expenses amount to between $100–$110 a month, leaving $23 (£2) a month for clothes, uniform, pocket-money, and general expenses, including ricksha hire. The item "drinks"—may seem to a casual observer a large sum, but I would remind readers that the East is a thirsty place, all mineral waters are dear, and stimulants are occasionally necessary. One cannot depend on the servants to boil the water regularly, and indeed it is generally spoken of by the faculty in the East as better left alone, or used for bathing purposes only.

SOCIAL LIFE.

As to social life, with the exception of the medical staff, hardly a soul displays the slightest degree of interest in either the nurses or their work. That there is such a thing as a hospital in their midst is only recognised when one of their own persoual friends is taken ill. That patients obliged to remain in bed should require literature or flowers to lighten the Tropical day, rarely occurs to them. Social life for a nurse is practically *nil*. If, in your hours of leisure you go amongst the people, they are kind; when you return to your hospital, you are buried. Even "invitations" to afternoon tea are few and far between. But, thank goodness, there are a few exceptions, and one does meet with women who, having a recognised position, see how hard and wearisome it is for girls living and working by themselves in a strange country, and who attempt to brighten their lives. One misses also one's own kind with whom to "hob-nob" and exchange ideas. In fine, I fear that the girl who will devote herself to nursing in this part of the East, must first realise that she will stand apart in the working world.

### Everybody's Opinion.

[Correspondence on all subjects is invited, but we cannot in any way be responsible for the opinions expressed by our correspondents. No communication can be entertained if the name and address of the correspondent is not given, as a guarantee of good faith but not necessarily for publication, or unless one side of the paper only is written on.]

### A QUESTION OF IMPORTANCE.

"JUSTICE" writes: I am anxious to know whether it is fair to the public and to nurses that nurses who are taken at a general hospital for three years' training and certificate should receive that certificate duly signed, when, in reality, they have only had two years in the wards and the third year are sent out on the private nursing staff, where, of course, they receive no further training, but are sometimes needed for occasional help in the wards. Can gaining experience only in their work be considered training?

### NOT ALLOWED TO THINK.

"A. M. D." writes: In my probationer days in hospital, to impress upon us the duty of implicit obedience to the orders of our superiors, I have known our sisters to tell us that we *must not think*—we must do as we were told. It got to be quite a by-word amongst the nurses—"You know very well that you mustn't think!" One day the charge nurse in one of our male wards overheard an amusing conversation between the two patients in beds Nos. 1 and 2. No. 1: "Why did ye get yer mither t' bring up yer claes to-day, Jack? Ye're no' goin' t' get up, are ye?" No. 2: "Aye, a thocht as they'd let me get up for a wee bit." No. 1: "Well, ye've nae richt t' think. D'ye no' ken, there's naebody allo'ed t' think here but the cook and the superintendent?"

### THE EXAMINATION QUESTION FOR JUNE.

"NURSE B. M. O." writes:—In connection with your examination question for this month I should like to tell you the following story: One day, walking in the wilds of a moorland district, we came upon a little stream, and, sitting on the bank, one leg carefully settled in the water, was an old man. It was a case of waiting for the "healing of the waters" if you like. There he sat; the vile, ulcerous leg with the clear, pure water rippling over it, was the truest irrigation the poor old man could have. He said he sat like that "most all the summer" and what good he got lasted through the winter! I may add that a little further down the stream was utilised as a cold storage for bottles of sterilised milk! and what other uses it was put to further on we did not explore.

### UNNECESSARY WORK FOR NURSES.

"SPIDER" writes:—Yes, it certainly is the rule for nurses trained in the leading London hospitals to have brasses to clean, tanks to polish, and ward linen to mend, and I don't think it hurts any of them. I myself was trained in a leading London hospital, and during the time I was probationer I had the "unnecessary work" to do which "Progress" complains of. And though I did not much relish it at the time, being a woman gently nurtured and one to whom such work is foreign, still I consider that such work is very good discipline. And I did not find that my hands got coarse either, as I took a moderate amount of care of them, to counteract the influence of soda, globe polish, and soft soap, besides that of the various disinfectants and antiseptics used.

"A. M. W." writes: I read with much interest the letter by "Progress" last week. I was trained in one of the leading London hospitals. There we certainly had to sweep, dust, clean out cupboards, scrub sinks, and polish a few "brights," an inkstand and lamp perhaps. Our menial work was only such as came into immediate contact with the patient. Surely a nurse knowing the evil of dust is far more likely to sweep the ward free of it than a wardmaid who has no more intelligent interest in her duties than a machine. Again, surely the sinks we scrub (not polish), which are exclusively for the patients' use, should not be relegated for cleanliness to the wardmaid. Thus the menial work was not excessive; it did not necessitate great physical exertion, and was chosen as part of the nurse's work for the benefit of the patient. However, in course of time I went to a provincial hospital, and my experience was very different from that of "Progress." Every morning numberless brasses had to be polished, sinks polished with turpentine, &c., once a week furniture polished, windows cleaned, and dados washed. The last-mentioned has recently been the wardmaid's work, in my opinion a mistake, as the nurse, recognising the necessity for absolute cleanliness, would clean the dados which come in close proximity to the patient to more purpose. The sweeping also was the wardmaid's work, with the invariable result that beds, lockers, &c., remained stationary with an accumulating mass of dust behind. Personally I would rather sweep the ward, wash dados, and have it wholesome than spend much physical exertion on brasses that benefit no one. "Progress" is right; the patients must suffer from the unnecessary brass polishing done by nurses. I have polished and polished without rhyme or reason for the sake of a smart and showy ward, with the result that, when the routine polishing is done, we are fit for nothing, and the patients get routine attention from a tired and silent nurse. Many of the nurses break down, not from the nursing they have done, but from the excessive physical strain of unnecessary work. Gently nurtured women find this work their chief difficulty: they are willing enough, but from experience know neither the quickest nor best methods of brass and sink polishing. Without doubt much unnecessary work is being done by nurses, but not exclusively in the London hospitals, and it does indeed seem a pity that a nurse's best work and thought should be expended on the ward furniture.

### THE NURSES' CO-OPERATION.

"An Old Member of the Nurses' Co-operation" writes: I am very pleased to hear that the nurses are to be allowed to call a meeting on the subject of the resignation by Miss Hughes of her post as lady superintendent. Those who have belonged to the Staff, and have sought other work, cannot help still feeling a great interes t in the institution and in the members. I hope that the loyalty of one and all—both of the nurses, of those who have been our staunch friends in the past, and of those also at present in office—will come out all the brighter for the brief time of anxiety. The keen and searching inquiry into all the money and business transactions of the Co-operation which must be made should ensure its satisfactory and efficient working in the future. Loyalty, sincerity, and watchfulness must be ever the watchwords, and I think that has been proved in the past. But it seems to me that to keep up to the rules which were formed in its early days, the nurses should be allowed a voice in any important matter before any decision is accepted or decided upon. That such has not been the case is to be regretted; but probably the meeting this week will clear up many matters which at present are obscure. All the members were much astonished to find that Miss Hughes had taken such an extreme measure, and those who have the pleasure of knowing her cannot help feeling that something very serious must have occurred to lead her to take such a step. The greater number of the staff hope that she will consent to remain lady superintendent for many years, and that those who have a right to do so will vote that Miss Hughes should be met, certainly half way, and that if possible anything not agreeable to her should be remedied. Should it be a matter of health which causes her retirement, a locum might be appointed at once, and so release Miss Hughes for a change, thus still securing the services of one who is respected and honoured by many.

### AN OPEN-AIR BABY.

"Nemo" writes, alluding to the letter in our issue for May 25 with the above heading: As I frequently have to nurse open-air babies in London and the suburbs, I think I should like to give you a short account of one just seven weeks old, who has been hand-fed. During April, although a few days were hot, the weather was by no means settled. In spite of this my baby was out at three days old and enjoyed to the fullest extent the fresh air; the bedroom windows were always open night and day, and when the wind was in the right direction the door also. Baby's cot, of course, was placed out of the draught, and the patient covered according to the warmth of the room. With regard to a hot water bottle I may mention that if baby's feet are cold I apply one at once, as cold feet will give a baby stomach-ache; the head should always be left free, as if covered the heat is liable to produce red gum (strophylus). My little charge was bathed freely twice each day, friction with the hand being performed all over the child after each bath before being dressed, and the clothes were all of flannel with a woollen vest, put on very loosely. It was then fed with cow's milk diluted with boiled water (one part milk, three parts water); this was continued for three weeks every two hours—sleeping or waking—during the day and every six hours during the night. Frequently I have raised baby asleep, fed her, and laid her down again and she would continue to sleep. When going out, as of course she did daily, she would sleep the whole time. After the three weeks the food was increased to half milk half water, and she was fed every four hours. With regard to the nurse in Florence letting her baby sleepfrom ten to eleven hours, I conclude that the child was over three months old. If younger a baby's digestive organs should not be allowed to go longer than six hours without food. Reverting to the bath, the baby enjoyed the water to the fullest extent and waited with delight for the water to be thrown over her, and when just over a fortnight old smiled and crowed if talked to. I am expecting shortly to nurse another baby who will, if it lives be brought up as an open-air baby, and I think and hope that this hygienic plan of working will become more universal.

### Appointments.

Burton-on-Trent General Infirmary.—Miss Ellen Mawson has been appointed night sister. She was trained at the Poplar and Stepney Sick Asylum for three years, and has since been extra nurse at the Edinburgh Royal Infirmary, and sister at the Poplar and Stepney Sick Asylum.

Chalmers Hospital, Banff.—Miss J. M'Kerrow has been appointed matron. She was trained at Charing Cross Hospital, London, and has since been night superintendent and assistant matron at the Royal Albert Edward Infirmary, Wigan.

Dorking Workhouse Infirmary.—Miss A. F. Shedden has been appointed superintendent nurse. She was trained at Birmingham Workhouse Infirmary, and has since been head maternity nurse at Hackney Union Infirmary.

Kettering Workhouse.—Miss Ellen Kavanagh has been appointed temporary night nurse. She is a member of the Meath Workhouse Nursing Association.

Mercers' Hospital, Dublin.—Miss Mary Turttle has been appointed sister of the theatre and men's surgical wards. She was trained at the Royal City of Dublin Hospital, and has since been temporary sister in the same institution, private nurse for the City of Dublin Nursing Institution, temporary sister and temporary night sister at the Mercers' Hospital.

Monkwearmouth and Southwick Hospital, Sunderland.—Miss Isabella Shields and Miss Lilian Burgess have been appointed staff nurses. They were both trained at the Monkwearmouth Hospital.

North Riding Infirmary, Middlesbrough.—Miss G. Waters has been appointed ward sister at the above infirmary. She was trained at the Leeds Union Infirmary and the Hove Isolation Hospital, Sussex.

Throat Hospital, Golden Square.—Miss Mary Elizabeth Brighton has been appointed staff nurse. She was trained at the North West London, Kentish Town, and has since been staff nurse at Shoreditch Infirmary.

Victoria Hospital, Folkestone.—Miss Winifred Plum has been appointed matron. She was trained at University College Hospital, and has been sister at the Crumpsall Infirmary, Manchester.

### Where to Go.

The Livingstone Exhibition.—This exhibition, which is practically a Travellers' Health Exhibition, opens at the Westminster Town Hall on Tuesday, June 18th. June 19th will be a special afternoon for nurses, when Robert Howard, M.B., will give some hints on nursing malaria.

### "The Hospital" Convalescent Fund.

The hon. secretary begs to acknowledge with many thanks the receipt of 5s. from Nurse H. S. Gibbons.

### Echoes from the Outside World.

### AN OPEN LETTER TO A HOSPITAL NURSE.

THOSE optimistic souls who feel hopeful that peace in South Africa is sure to come soon because of Mrs. Botha's visit to England are, I fear, doomed to disappointment. Although she has very wisely declined to see any journalists or to allow Mr. Fischer—the son of a former State Secretary of the Orange River Colony, and till last June a captain of one of the Free State scouting corps—who accompanies her, to say anything authoritatively on her behalf, it is, I think, obvious to all that it is impossible for the Government to treat seriously any overtures made to them in England by the wife of a commander holding no official position in the country which she is supposed to represent. Any efforts at restoring peace, to be successful, must, I should fancy, come to Lord Kitchener from those now in rebellion against him. But it is owing to the kindness of the Commander-in-Chief in South Africa that she is allowed to travel to Europe, and though her visit may be in no sense official, Mrs. Botha will not be the first woman, more particularly as she is a charming personality, well educated and always well dressed, who has by her influence played quietly and unostentatiously an important part in the affairs of her country. Mrs. Botha, who has brought her seven-year-old son with her, leaving her girls at a school in Natal and her eldest boy with his father, is far from well—the anxiety she has gone through is alone enough to account for ill-health, more especially if, as we are told, she has strained every nerve to persuade her husband to arrange for the end of the war—and left for the Continent on Tuesday.

IF Mr. Kruger will receive her, she contemplates going to see him. His new home, half an hour by train from Amsterdam, is a villa given him by a Dutch nobleman, furnished in strange style, nearly all the furniture having been presented to him by friends and admirers. Naturally the result may be comfort, but it is hardly likely to be elegance. Some of the gifts are especially handsome, and the incongruity of the odd assortment is very striking. Oom Paul has a staff of secretaries and retainers, the former all well educated men, knowing two or three languages. His habits are strange, but very methodical. He retires to rest every night at nine o'clock. At midnight he rises, and with his secretaries dictates and writes till two o'clock in the morning. Then he devotes a short time to reading his Bible, and finally returns to bed till the morning. This routine never varies, but is carried out most methodically. Mr. Kruger still states that he believes firmly in the success of the Boer arms and ultimate independence, but weeps when he speaks of his wife, because he fears that it will be so long before they meet, her health being too precarious for her to travel to Holland, and his too delicate to allow of his return to Africa. But his eyes seem well again, "as bright and as clear as a boy's," the trouble having been caused by a swelling beneath the eye rather than by any serious trouble with the orbs themselves. According to the account of his interviewer, he added pathetically, when speaking of the ultimate success of the Transvaalers, "I may not live to see the end, but there is another ready to take my place." One cannot help wondering what "his place" is now that he has fled from his land in her trouble and distress.

THE world lost three interesting personalities on Monday. Lord Wantage in his early days was a gallant soldier, and greatly distinguished himself in the Crimean War. For bravery at Inkermann he was awarded the Victoria Cross. In more recent times, since he exchanged the name of Colonel Loyd-Lindsay for that of Lord Wantage, he assisted to establish the National Red Cross Society, and was the much-esteemed chairman of the Council. He was very wealthy and extremely generous. Mr. Robert Buchanan, who passed away at Streatham after a lingering illness, was a poet of some repute and wrote two or three striking novels. His considerable abilities were by no means turned to the best account, and it cannot honestly be said of him that he materially enriched the literature of his country. The death of Sir Walter Besant will be widely mourned by the thousands who have enjoyed his delightful books, and rejoiced at the wholesome influence he exercised in the pursuit of his profession. As you may remember, he first distinguished himself as a collaborateur with James Rice, and when the clever pair had produced *The Golden Butterfly* they reached the zenith of their fame. Afterwards Sir Walter, who was knighted six years ago, made a great hit on his own account with *All Sorts and Conditions of Men*, which was followed by that charming story *All in a Garden Fair*, and a succession of more or less readable works of fiction. At the time of his illness, which assumed a serious form three weeks ago, Sir Walter was engaged in the congenial task of rewriting Stow's *London Survey*. He not only loved London, but knew all about it; and it was perhaps the greatest joy of his life when the dream of *All Sorts and Conditions of Men* was realised by the erection of the People's Palace at Mile End. Successful himself, he was always ready to assist struggling authors, notably Robert Louis Stevenson, and he leaves behind him an honoured name.

HAPPY the cycling nurse who has never yet had a severe spill! I am afraid there are few able to boast that they have had all the pleasure of a bicycle and none of the pain, for unfortunately it is not only the careless or foolhardy rider who comes to grief. On Sunday as I was going to church, a girl was proceeding slowly and carefully along the high road. Suddenly, from behind, a light trap approached, the driver's thoughts apparently elsewhere, for, without warning of any sort, he went straight on. In far less time than it takes to write the horse had touched the girl, knocked her off, and ridden over her. Luckily the cart did not pass over her body, and I did not stop to hear any details, plenty of help being available; but my last vision of the poor cyclist as she was extricated from under the horse's hoofs was sufficiently dreadful to make one very apprehensive of the severity of her injuries. The incident made me wish that someone with as much promptitude as the King of Denmark had been at hand. Although he is over eighty years of age he has still a quick eye and a ready hand. He was recently taking his daily promenade in the streets of Copenhagen with his son, Prince Waldemar, when he came in sight of a lady cyclist. At the same time a cab, driven at great speed, was coming by, and both the cyclist and the driver of the vehicle were so occupied with looking at their Sovereign that a collision occurred and the girl was unseated. In another second the horse's feet would have trampled upon her, but the King rushed forward and with one hand turned the horse aside, while with the other he drew the recumbent figure out of danger. How proud the girl must be to think that she has been saved from serious injury by her aged monarch! No wonder that the enthusiasm of the spectators of this little scene waxed so fervent that they cheered again and again.

MANY nurses would like to have been in Madame Sarah Bernhardt's shoes on Monday. Accompanied by a friend, she went down to the Houses of Parliament at the invitation of a member, and having been shown the Lobby was entertained at tea on the Terrace. The river looks particularly well seen from this vantage point, and most people admit that they had no idea till they had been on the Terrace that the Thames in the metropolis could appear so picturesque. After tea the famous French actress paid a visit to the House of Lords, and if there was any business going on would sit in the queer little boxes close to the entrance reserved for strangers, which remind one so strongly of the old-fashioned pews known irreverently as "horse-boxes." Later on she was escorted to the Ladies' Gallery in the Commons, and took her seat behind the Grill to listen to the discussion on the Bill to renew and increase the Civil List of the Sovereign, and saw the whole process of a division taken on one of the amendments moved in Committee on the measure. Some years ago it was comparatively easy to obtain access to the popular Chamber, but every week it becomes more difficult, and on any great occasion the life of a member who has many lady friends is said to be scarcely worth living.

### For Reading to the Sick.

RESTFUL DEPENDENCE.

LORD, I had planned to do Thee service true,
To be more humbly watchful unto prayer,
More faithful in obedience to Thy Word,
More bent to put away all earthly care.

Of time all consecrated to Thy will,
Of strength spent gladly for Thee, day by day—
When suddenly the heavenly mandate came,
That I should give it all, at once, away.

Thy blessèd hand came forth, and laid me down,
Turned every beating pulse to throbs of pain,
Hushed all my prayers into one feeble cry,
Then bid me to believe that loss was gain.

And *was* it loss, to have indulged such hopes?
Nay, they were gifts from out the inner shrine;
Garlands, that I might hang about Thy Cross,
Gems, to surrender at the call Divine.

As chiselled image unresisting lies
In niche by its own sculptor's hand designed,
So, to my unemployed and silent life,
Let me in quiet meekness be resigned.

*C. M. Noel.*

The *whole Discipline of Life* teaches us this same lesson of Dependence.

We see it in all the changes of outward life. How little we know what a day may bring forth—what may happen to us in our body, or what may happen as to our work; how it may be altered; how we may be taken from it, or it may be taken from us! Does not this make us depend necessarily upon a higher Being?

This, again, is the meaning of many of your *failures.* Go over your life: the good that you have tried to do, the souls that you have honestly tried to benefit. God has loved you and blessed you for it: and he is blessing you now, by letting you see the meaning of all the failures; how the health broke down, and the plans came to nothing, and the resolutions seemed only to end in hopeless defeat—how you have broken down in body, soul, and spirit—all because, in His tender love, God was teaching you to depend upon One outside of yourself. This was the meaning of all that strange mingling of trial and blessing, failure and success—partial success, to cheer you on, because God knew that you could not bear entire failure; partial failure, because entire success would have fostered pride and self-dependence.

He drank that cup of suffering, that He might know what *you* feel, when you are left alone; if people seem to be tired of you; if you no longer have the power to concentrate your mind on your reading and your prayers, as you once did. HE knows what it is, to feel helpless!

And it is this thought—of the Sympathy of CHRIST—which helps us, most of all, "to enter into that Rest." This it is, which nerves us to bear the discipline: to remain patient in the fire, while GOD is melting the old stubborn iron, that He may mould it into new and beautiful forms, fashioned after the likeness of the Blessed Ones in Paradise. This it is, which enables us to "labour to enter into that Rest;" it is not merely the thought that CHRIST suffered, and suffered more than we can suffer; but the thought that He is "the Same, yesterday, and to-day, and for ever:" that He remembers what He felt in His own sufferings, as Man; and knows the help which each sufferer will need, in each special trial.—*Bishop Wilkinson.*

### Notes and Queries.

**The Editor is always willing to answer in this column, without any fee, all reasonable questions, as soon as possible.**
**But the following rules must be carefully observed:—**

1. **Every communication must be accompanied by the name and address of the writer.**
2. **The question must always bear upon nursing, directly or indirectly.**

**If an answer is required by letter a fee of half-a-crown must be enclosed with the note containing the inquiry.**

*Certificated Monthly Nursing.*

(103) I should be glad to know the names and addresses of institutions in London where certificated monthly nurses are received at a fixed annual salary, the institution receiving fees.—*E. P. J.*

You do not say whether you have had midwifery training. But you might write to the Maternity Nursing Mission, 171A King's Cross Road, N.W., and the Royal Maternity Charity, 31 Finsbury Square, E.C.

*Homes for Phthisis.*

(104) Would you kindly tell me if there are any Sanatoria for Consumption where district patients could be sent on payment of a small fee weekly and with a subscriber's recommendation.—*District Nurse.*

Your patient is eligible for the National Hospital for Consumption, Ventnor, the Hospital for Consumption, Brompton, the North London Hospital for Consumption, Mount Vernon, Hampstead, N.W., and others. See also the "Sanatoria for Consumption" published in THE HOSPITAL of April 20.

*Training.*

(105) Will you kindly tell me where I can get training in general and monthly nursing without paying a premium. I could give a year's probation without salary. My age is 31.—*Ellen B.*

You might get what you want in a workhouse infirmary, or in a small nursing home. We should recommend you, however, to obtain a three years' certificate, in which case you could earn a salary through the whole period of your training.

*Rheumatism.*

(106) Would you kindly give me the address where hot-air baths and electricity for the cure of rheumatism may be had in London.—*Anxious.*

Apply to the Dowsing Radiant Heat and Light Treatment, 24 Budge Row, London, E.C., or to Mr. Tallerman, 50 Welbeck Street.

*Gynæcology.*

(107) 1. Please recommend a good book on gynæcological nursing.
2. Are the Royal Infirmary and the Union Infirmary, at Bradford, separate institutions?—*Training.*

1. You will find "Gynæcological Nursing," by G. A. Hawkins-Ambler, F.R.C.S., excellent. Price 1s. from the Scientific Press. 2. Yes.

*Massage.*

(108) Will you tell me how I can study massage without paying much money. I am a certificated nurse.—*Annie H.*

As you live in London you could perhaps arrange to attend the classes at the National Hospital for the Paralysed and Epileptic, Queen's Square, Bloomsbury, W.C.

*Young Pro.*

(109) Can you tell me if there is any infirmary or cottage hospital where a girl of eighteen can be trained as nurse?—*Mary.*

She is too young for any of the general training schools. You might hear, by advertisement, of a small home where she would be taught something of nursing, but the teaching would be little valued in the nursing world.

*Nauheim Baths.*

(110) Would you kindly tell me the method of giving Nauheim Baths with circulatory movements in the patient's house.—*E. L.*

You will find "Medical Gymnastics, including the Schott (Nauheim) Movements" useful. Price 2s. 6d. from The Scientific Press.

*Matron.*

(111) I have been head nurse of a Crèche for ten months, do you think I can get a situation as matron of a children's home, convalescent or otherwise?—*Agnes W.*

We cannot possibly say; you can always try by advertising and by answering advertisements.

*Army Nursing Reserve.*

(112) I wish to become a member of the Army Nursing Reserve (could you give me the address), or failing this, I want to get an appointment abroad. Would you kindly tell me where to apply.—*Patie C.*

The address is 18 Victoria Street, S.W., and you might possibly obtain some appointments through the Colonial Nursing Association, Imperial Institute, S.W.

*Johannesburg.*

(113) Can you give me any particulars of Johannesburg Hospital? I want to know how to get on to the nursing staff, and also the prospects of private nursing.—*G. E. M.*

You should apply to the Nursing Superintendent, Johannesburg Hospital (Transvaal), address P.O., Box 1050. The nursing staff consists of 27 nursing sisters, 23 lay nurses, and 27 probationers.

**Standard Books of Reference.**

"The Nursing Profession: How and Where to Train." **2s. net; post free 2s. 4d.**
"Burdett's Official Nursing Directory." 3s. net.; post free, 3s. 4d.
"Burdett's Hospitals and Charities." 5s.
"The Nurses' Dictionary of Medical Terms." **2s.**
"Burdett's Series of Nursing Text-Books." **1s. each.**
"A Handbook for Nurses." (Illustrated.) **5s.**
"Nursing: Its Theory and Practice." **New Edition. 3s. 6d.**
"Helps in Sickness and to Health." **Fifteenth Thousand. 5s.**
"The Physiological Feeding of Infants." **1s.**
"The Physiological Nursery Chart." **1s.; post free, 1s. 3d.**
"Hospital Expenditure: The Commissariat." **2s. 6d.**
**All these are published by THE SCIENTIFIC PRESS, Ltd., and may be obtained through any bookseller or direct from the publishers, 28 and 29 Southampton Street, London, W.C.**

### Travel Notes.

By Our Travelling Correspondent.

### LXXVII.—ROUND AND ABOUT GLASGOW.

There is so much to be seen in the neighbourhood of Glasgow that the only difficulty is to make a selection. The city lies close to much of the finest scenery in Scotland and a great deal of it can be visited in single-day excursions, so as not to incur the expense of shifting your sleeping quarters. I think it would interest you to refresh your memory of Sir Walter Scott's works, both prose and poetry, before visiting Scotland. "Old Mortality," "Rob Roy" and "Waverley" have their scenes laid in the part of the Western Highlands that we are now considering, also "The Lord of the Isles" and "The Lady of the Lake."

### The Trossachs, Loch Lomond, and Loch Katrine.

These can all be seen in one day and the cost is under £1. Of course it would be much pleasanter to remain one or two days at one of the many beautiful spots in this district, say Inversnaid or Aberfoyle, but that runs up cost at once, and it can be done otherwise if desired. Trains, steamers, and coaches are all arranged to connect without difficulty. Every turn of the road reveals fresh beauties and it is hard to say where to locate the loveliest spot.

Coming from Glasgow you take train to Balloch, and there get into the Loch Lomond steamer. Shortly after leaving Balloch you see Arden to the west, and immediately behind is Glen Fruin, full of sinister memories of the implacable feuds of the Highland clans. The name signifies the Glen of Sorrow. Here in 1592 the chief of the Colquhouns was murdered, and later a furious battle was fought between the Colquhouns and the MacGregors, the latter coming off victorious after almost annihilating the Colquhouns.

Rowardennan is the best point for ascending Ben Lomond, 3,192 feet; but the idly-minded can go all the way on a pony, if so disposed. I need hardly say that if you contemplate this effort at mountaineering it cannot be done in a day's excursion. At Inversnaid you take the coach for Loch Katrine and the Trossachs. The silver strand on Loch Katrine is not so lovely as it was in Scott's time, because the water level has in some way been raised by the Glasgow Water Corporation and so it is almost submerged, but the place remains wonderfully beautiful and a sight that one never forgets. Towards evening is the best time for the view of Ben Venue seen from Loch Katrine.

The pass which goes by the name of the Trossachs, is about two miles long and very grand.

### The Falls of the Clyde.

You take the train to Lanark, in itself an interesting old town, and from there visit Corra Linn and Bonnington; a third fall, that of Stonebyres, lies in a different direction, and is not so fine. Corra Linn is the grandest of all. On the same day you might visit Craignethan Castle, which Scott calls Tillietudlem in "Old Mortality." As we enter the grey old ruins we see before us Lady Margaret Bellenden with her "black scarf and manteau" going to meet Francis Stuart Bothwell; and entering the dismantled hall we hear again the tedious and oft-repeated account from the old lady's lips of how "his sainted Majesty took his disjune here," and we gaze in spirit upon that delightful scene when Edith, overpowered by hearing of the bad news concerning Henry Morton, sinks into the embrace of the armchair once honoured by royal occupation, to the horror of Jenny Denison. "Oh! Miss Edith; haud your heart up about it, it's maybe no true, for a' that I hae said . . . What i my Leddy comes? or the Major?—and she sitting in the throne too, that naebody has sat in since that weary morning the King was here!"

Not far off is Bothwell Brig, some two miles from Glasgow where Monmouth defeated the Covenanters in 1679.

### The Kyles of Bute.

There are numerous excursions round Bute, Cantire, Arran, and Argyll. Steamers go every day; the fares are very low and the scenery is magnificent. One very nice day's trip is in the *Columba* or *Iona*. You pass Rothesay, then through the Kyles, driving across to Otter, and rejoining the steamer and proceeding up Loch Fyne.

In connection with this trip is a prolongation through Loch Awe up to Dalmally and home by rail. It is astonishing how much ground can be covered by the very judicious co-operation of the steamboat and railway companies.

### Inverary, the Seat of the Argylls.

The *Lord of the Isles* makes this trip every day and an hour is allowed for seeing the town and castle. The latter is not architecturally interesting and only dates from 1744; moreover, much of that first building was destroyed by fire in 1877. It is only in the absence of the family that it is shown. We read much of how the scenery struck Scott in the "Legend of Montrose." Captain Dalgetty was always occupied in considering how the country and the different castles should be fortified to the best advantage. The old stronghold of the Campbells was Kilchurn Castle on Loch Awe; this caused the slogan of the clan to be, "It's a far cry to Lochowe," meaning to hint at the extreme difficulty of dealing with the family in their mountain fastnesses.

### Ardrossan and Largs.

Another day you may go to Wemyss Bay by train and take a steamer to Largs and then on to Ardrossan, which is rather an important seaport and from which ocean-going ships sail to Ireland and the Isle of Man. Continuing down the coast you come to Ayr, of no particular interest except as it is connected with Burns. He was born about two miles from Ayr, and one can trace the sites of the different incidents in his works round about. Very near is "Alloway's auld haunted kirk" and Mungo's Well. The Brig o' Doon, too, is worth a visit. There are many more excursions of which I have no time to tell you, but almost all are easily managed in one day.

### TRAVEL NOTES AND QUERIES.

Boarding House in Paris (E. P.).—You give no pseudonym, and your question is very confused. I am not sure I understand what you want. Second-class return ticket to Paris is £2 2s. 3d., and she can get accommodation for another £2 for the week at Mrs. Summers, 226 Boulevard Raspail, also, I think, at Miss Worthington, 4 Rue des Pyramids. Write beforehand, as these kind of pensions often change hands. Ostend is quite out of the question, being extremely dear. Second return to Bruges £1 19s. 1d. Pension for £2 at Hôtel Panier d'Or, in the market place. Probably there are English pensions in Bruges, but I do not know them. English is not so much spoken as in Paris. If I knew rather more of your needs I could help you better. Write again and tell me more.

Normandy, Brittany, or Belgium (Nurse Mary).—You would do well to go to Antwerp from Leith and then on into the Ardennes, where the scenery is charming. Expense, second-class rail return to Dinant from Antwerp, about 16s. At Dinant stay at the Tête d'Or: terms 8 francs per day, which would be £2 5s. per week. Thus three weeks would come to £6 15s.; tips, &c., to another £1; and your rail to 16s., making £8 11s. Brittany would be very pleasant, but there is no way of saving the long journey down to Southampton, which swallows up so much money. However, you might like a week at St. Servan and St. Michel and a week at Morlaix and another at Paimpol, or somewhere about there. It would come more expensive because you have not the help of the voyage from Leith. I fancy Belgium is the best for you. I conclude you are not going alone, and might like to have a glimpse at some of the old Flemish cities. If not alone I could give you names of cheaper accommodation at Dinant. Tell me if I can help you further. Normandy is more expensive.

After Cure in the Black Forest (Nemo).—After a course at Schwalbach some patients are obliged to visit Schlangenbad for a short course, on account of a roughness of skin which attacks some from the Schwalbach waters; this effect, however, is not frequent. There are several places that may be chosen for the after cure, such as Freudenstadt-Badenweiler, &c.; but you should not settle upon any place without your doctor's advice. He may prefer Switzerland for many reasons.

South Brittany (Cyclist).—The best plan will be for you to send your luggage on by diligence when there is one, or if the distance is not great take a third-class ticket as if travelling yourself; your things will then go quickly as passenger's luggage, and you can claim them when you arrive in the evening. This plan is good for short distances, and obviates tedious waiting. Brittany is a country slow in its movements, and impedimenta forwarded by luggage train has a pleasant way of delaying its appearance. Quiberon is quite within a day's run from Auray; if you start early you need not hurry or feel over-fatigued, returning before dark. It is a pity you cannot start earlier in the summer, so as to have the benefit of the long light evenings. I think you would do well to go viâ Rennes and start cycling from Vannes, a most quaint town, taking the line of Quimperle-Quimper-Landerneau-Morlaix-Lannion and Perrosquiree, and so on round to St. Malo and home by Southampton.

